# Anti-carbamylated protein antibodies precede disease onset in monkeys with collagen-induced arthritis

**DOI:** 10.1186/s13075-017-1455-1

**Published:** 2017-11-02

**Authors:** Marije K. Verheul, Michel P. M. Vierboom, Bert A. ’t Hart, Rene E. M. Toes, Leendert A. Trouw

**Affiliations:** 1 Department of Rheumatology, Leiden University Medical Center, Postbus 9600, 2300 RC Leiden, The Netherlands; 2 Department of Parasitology, Biomedical Primate Research Centre, Rijswijk, The Netherlands; 3 Department of Immunobiology, Biomedical Primate Research Centre, Rijswijk, The Netherlands; 4 Department Neuroscience, University of Groningen, University Medical Center, Groningen, The Netherlands; 5 Department of Immunohematology and Blood Transfusion, Leiden University Medical Center, Leiden, The Netherlands

**Keywords:** Collagen-induced arthritis, Rheumatoid arthritis, Autoantibodies, Anti-CarP antibodies, Rhesus monkeys

## Abstract

**Background:**

Rheumatoid factor (RF), anti-citrullinated protein antibodies (ACPA) and anti-carbamylated protein (anti-CarP) antibodies are rheumatoid arthritis (RA)-associated autoantibodies. Besides their presence in human serum, anti-CarP antibodies have also been described in rodent models of arthritis, while ACPA are not consistently detectable. Data on these RA-associated autoantibodies in primates are not available. Therefore, we investigated the presence of RF, anti-CarP antibodies and ACPA in rhesus monkeys before and after collagen-induced arthritis immunizations.

**Methods:**

In previous studies, arthritis was induced in groups of rhesus monkeys by immunisation with collagen following pre-treatment with placebo, abatacept or Roactemra. Previously collected serum was used to measure, autoantibodies by ELISA, detecting anti-CarP antibodies, RF-IgM and antibodies against CCP2, citrullinated myelin basic protein and citrullinated fibrinogen.

**Results:**

Out of the three autoantibodies, only anti-CarP antibodies were detectable in resus monkeys with arthritis. RF-IgM and ACPA were undetectable and below the detection limit of the ELISA. The level of anti-CarP antibodies increases over time and, similar to in humans and mice, these autoantibodies were already detectable before clinical disease onset. Furthermore, preventive treatment with abatacept (CTLA4/IgG1-Fc fusion protein) inhibited the development of anti-CarP antibodies after immunization, while this was less evident for preventive Roactemra (anti-IL6-receptor) treatment. Moreover, disease progression was only reduced following abatacept treatment.

**Conclusion:**

Rhesus monkeys develop anti-CarP antibodies upon induction of collagen-induced arthritis, while we were unable to detect RF or ACPA. Also, the development of anti-CarP antibodies could be inhibited by preventive abatacept treatment.

## Background

Rheumatoid arthritis (RA) is a chronic autoimmune disorder characterized, among others, by the presence of autoantibodies. The most well-known autoantibodies in RA are rheumatoid factor (RF) and anti-citrullinated protein antibodies (ACPA). RF, which recognizes the IgG Fc region, was the first to be discovered. This autoantibody occurs in approximately 70% of patients with RA but has rather low specificity (79%) when compared to healthy controls [1]. ACPA target citrulline residues, which arise after the enzymatic conversion of an arginine residue by peptidylarginine deiminases [2]. When compared to RF, ACPA are thought to have higher specificity (up to 98%) and can be detected in up to 69% of patients with RA [1]. Therefore, both ACPA and RF have been incorporated into the classification criteria for RA [3] and are often used for diagnostic purposes.

Other autoantibodies have been identified more recently [4], including anti-carbamylated protein antibodies (anti-CarP) [5]. These antibodies target carbamylated proteins in which a lysine has been converted into a homocitrulline under the influence of cyanate. Anti-CarP antibodies have been detected in serum in up to 46% of patients with RA in several cohorts around the world and are shown to be associated with the development of joint damage [5–10]. These antibodies could be detected several years before disease onset and are associated with development of RA in patients with arthralgia [6, 11, 12]. Furthermore, anti-CarP antibodies recognize multiple carbamylated antigens, such as human serum albumin, fibrinogen and alpha-1 antitrypsin [5, 13–15]. Although many clinical associations have been identified for all three of the autoantibodies discussed, their role in the pathogenesis of RA is still under debate.

Anti-CarP antibodies have not only been detected in human RA, but also in different rodent models of arthritis [16, 17]. RF-like antibodies have also been described in both rodents and human [18]. While the presence of ACPA is evident in RA, they are largely undetectable in rodents [3, 5, 16, 19]. The combination of these autoantibodies has not been measured in primates yet. We have used the collagen-induced arthritis (CIA) model in rhesus monkeys to establish the occurrence of these autoantibodies in arthritis This model was previously established for the investigation of RA and a large number of clinical parameters have already been established [20].

In order to investigate RA-associated autoantibodies in the primate CIA model, we made use of samples collected previously in prior studies that used this CIA model. We first determined the presence of ACPA, RF and anti-CarP antibodies. Second, we investigated the effect of two different treatments, Roactemra (anti-IL6-receptor) and abatacept (CTLA4-IgG1 Fc fusion protein) on the clinical score and autoantibody development after immunization.

## Methods

### Animals

This study was conducted at the Biomedical Primate Research Centre (BPRC; Rijswijk, the Netherlands) in accordance with the Dutch law on animal experimentation. The study protocol and experimental procedures were approved by the Experimental Animal Care and Use Committee of the BPRC. CIA-susceptible adult, healthy rhesus monkeys (*Macaca mulatta*; BPRC) were selected based on the absence of the dominant major histocompatibility complex class I resistance marker Mamu-B26 [21]. Individual data are shown in Tables 1 and 2.

**Table 1:** Animal identification, gender, age and starting weight at day of stratification

Study	Treatment	Animal ID	Gender	Age	Starting weight
1	Group I (placebo)	95020	M	14.8	10.2
		R05029	M	4.9	6.1
		R05053	M	4.8	7.8
		R05058	M	4.8	7.3
		R05073	M	4.8	6.6
	Group II (Roactemra)	95031	M	14.8	12.7
		BB226	M	6.8	9.9
		R04042	M	5.9	7.5
		R05059	M	4.8	6.9
		R05061	M	4.8	7.2
		R05089	F	4.8	5.1
		R05090	M	4.7	8.0
2	Group III (placebo)	R00062	F	12.2	5.2
		R02049	F	10.3	7.7
		R05068	F	7.3	5.9
		R06045	M	6.3	7.2
		R07111	M	5.2	5.6
	Group IV (abatacept)	96089	F	16.3	8.5
		R01091	F	11.1	5.0
		R05084	F	7.3	6.9
		R07003	M	5.5	6.5
		R07031	F	5.4	4.6
		R07068	M	5.3	7.6
		R07075	F	5.3	5.2

*M* male, *F* female

**Table 2:**
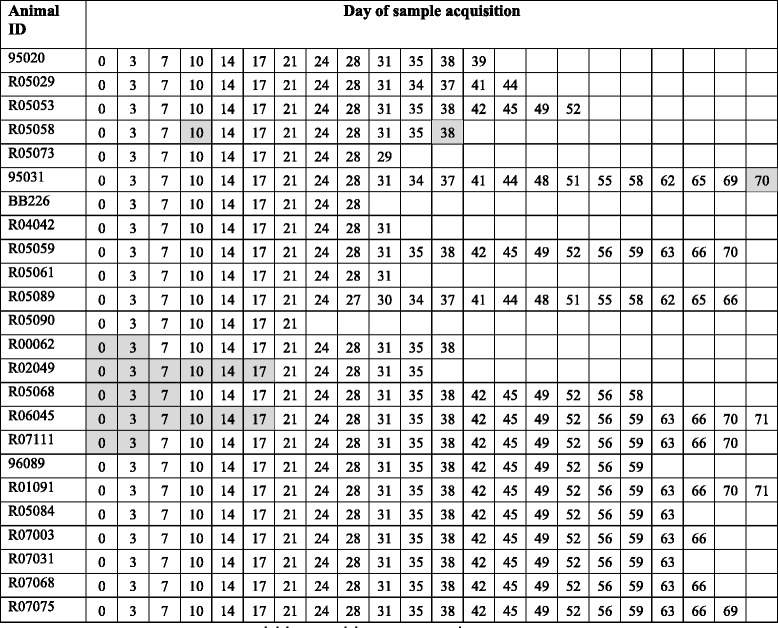
Day of sample acquisition specified for each animal. Samples that were not available for measurement are depicted in grey

### CIA induction

CIA was induced by immunization with chicken type II collagen (MD biosciences) as before [22, 23]. The collagen was dissolved in 0.1 M acetic acid to a final concentration of 10 mg/ml and mixed with an equal volume of complete Freund’s adjuvant (CFA; DIFCO). This emulsion was injected intra-cutaneously and distributed over 10 spots of 100 μl on the back of the animal, resulting in a final dose of 5 mg of chicken type II collagen per animal.

### Treatment conditions

The data were obtained from two studies reported elsewhere [22, 23]. The first study [22] contained a group that was placebo-treated (n = 5) and a group that was Roactemra-treated (10 mg/kg; n = 7) group. Roactemra or placebo treatments were given weekly, starting 7 days after immunization until day 42 and were administered as intravenous (i.v.) bolus injections. The second study [23] contained a group that was placebo-treated (n = 5) and an abatacept-treated (n = 7) group. Abatacept (10 mg/kg) and placebo treatment were administered starting from day 0 till day 42 as i.v bolus injections. (The ethical permits for these studies were obtained under number #633 and #695).

### Clinical evaluation

For clinical and ethical management, signs of clinical arthritis, soft tissue swelling (STS) and redness of affected joints were scored twice weekly using a previously published semi-quantitative clinical score [24]: 0 = no disease symptoms; 0.5 = fever; 1 = apathy and loss of appetite and weight loss; 2 = warm and tender joints, but without STS; 3 = moderate STS but normal flexibility of affected joints; 4 = severe STS with joint stiffness; 5 = such severe disease that euthanasia is necessary. Note that scores 0.5, 1 and 2 are sub-clinical CIA scores; CIA scores ≥3 represent clinically evident arthritis. To ensure objective clinical scoring the investigators performing the physical examination and rating clinical scores were blinded to the different treatments during the in vivo part of the study.

### Measurement of anti-CarP antibodies

The samples used for anti-CarP antibody measurements were stored previously in 100 μl aliquots at −80 °C. Anti-CarP-IgG antibodies were measured as described before, using carbamylated fetal calf serum as the antigenic target [5]. ACPA were measured using an in-house assay with CCP2 peptide, citrullinated fibrinogen (cit-fib, Sigma) or myelin basic protein (cit-MBP, Sigma) as the antigen. For each antigen, both the modified and non-modified versions were taken along.

For CCP2, streptavidin (Invitrogen) was coated on a plate at 5 μg/ml in a carbonate buffer with a pH of 9.6 and incubated at 4 °C overnight. Peptides were coated at 1 μg/ml in PBS with 1% BSA (Sigma) and allowed to bind for 1 hour at room temperature. Serum samples were diluted × 50 in PBS with 1% BSA and 0.05% Tween (Sigma) and incubated for 1 hour at 37 °C.

For Cit-Fib and Cit-MBP, the proteins were coated at 10 μg/ml in a 9.6 pH carbonate buffer and incubated overnight at 4 °C. Plates were blocked for 1 hour at 37 °C with PBS 2% BSA, pH 9.0. Samples were diluted × 50 in radioimmunoassay (RIA) buffer and incubated for 1 hour at 37 °C.

For the anti-CarP and anti-citrulline ELISAs, antibody binding to the target was detected using rabbit anti-human IgG-HRP(DAKO, P0214), which is also cross-reactive for the IgG antibodies from rhesus monkeys.

RF-IgM antibodies were also measured with an in-house ELISA, using rabbit IgG (Sigma) as the antigen, which was diluted to 10 μg/ml in carbonate buffer, pH 9.6 and incubated overnight at room temperature. Plates were blocked for 1 hour at 37 °C with PBS, 1% BSA. Serum samples were diluted × 100 in PBS, 1% BSA and 0.05% Tween and incubated for 1 hour at 37 °C. Antibody binding was detected with goat-anti-human-IgM-HRP (Life technologies, 627520). Cross-reactivity of this antibody was confirmed by measuring anti-collagen IgM antibodies in a selection of the monkey serum samples. All washing steps were carried out with PBS, 0.05% Tween. 2,2′-azino-bis(3-ethylbenzothiazoline-6-sulphonic acid) (ABTS) was used for final detection.

### Statistics

Statistical analyses were carried out in SPSS statistics version 23 (IBM) or with Graphpad Prism version 7. To compare antibody levels over time within the same animals, the Wilcoxon rank test was carried out. To compare antibody levels between two groups, the Mann-Whitney U test was carried out. Spearman correlation was tested to investigate a possible association between autoantibodies and clinical parameters.

## Results

**Fig. 1:**
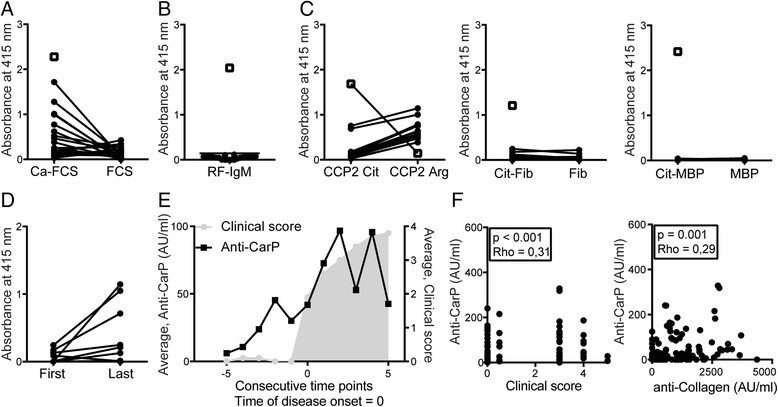
Anti-carbamylated protein (anti-CarP) antibodies can be detected in rhesus monkeys, while anti-citrullinated protein antibodies (ACPA) and rheumatoid factor (RF) cannot. **a** Anti-CarP IgG antibodies were measured by ELISA. Carbamylated fetal calf serum (Ca-FCS) and the non-modified fetal calf serum (FCS) served as the (control) antigen. The open square indicates a human positive control and the last two available primate samples for each monkey are indicated in closed circles. **b** RF-IgM antibodies were measured by ELISA, using rabbit IgG as binding antigen. The open square indicates a human positive control and the last two available primate samples for each monkey are indicated in closed circles. **c** ACPA IgG antibodies were measured by ELISA, using several citrullinated and control peptides or proteins as antigens. The open square indicates a human positive control and the last two available primate samples for each monkey are indicated in closed circles. **d** Anti-CarP IgG antibodies are shown for both the first available sample, taken at the time point of immunization and the last available sample in comparison to each other. Antibodies were measured as in **a**. The absorbance values for the FCS were subtracted from the Ca-FCS absorbance values. All samples were measured on one plate. ELISAs were carried out using the 10 control monkeys that did not receive any (preventive) treatment (**e**). Anti-CarP IgG antibodies and clinical score are compared over time. The point of disease onset was set at 0. Disease onset was defined as the point at which the clinical score was higher than 0.5 and increased at the next time point. Also, a persistent clinical score >3 had to be present as well. Therefore, three monkeys had to be excluded from this analysis. The grey area indicates the average clinical score, while the black circles show the average levels of anti-CarP antibodies at that particular time point. **f** Correlation between anti-CarP antibodies and clinical score or anti-Collagen IgG antibodies was investigated. All time points in all 10 monkeys were included for this analysis. The presence of possible associations was tested by spearman correlation. CCP2, cyclic citrullinated peptide 2; cit, citrullinated; arg, arginine control; Fib, fibrinogen; MBP, myelin basic protein; AU/ml, arbitrary units per millilitre; ELISA, enzyme-linked immunosorbent assay

The presence of RA-associated autoantibodies was measured in 10 rhesus monkeys at the last two available time points after CIA induction. Anti-CarP antibodies were readily detectable (Fig. 1a), while RF was not observed (Fig. 1b). ACPA were measured using three different antigens; CCP2 peptide, Cit-fib and cit-MBP, but no specific citrulline-directed signal could be detected (Fig. 1c). In summary, of the three investigated RA-associated autoantibodies, only anti-CarP antibodies but not ACPA or RF were detected in monkeys with CIA. Therefore, we further investigated the development of anti-CarP antibodies. A comparison of the anti-CarP antibodies between the time of immunization and the last available time point shows that the levels of anti-CarP antibodies increase over time (Fig. 1d). Furthermore, we observed that the anti-CarP antibodies are present before disease onset (Fig. 1e). Weak correlation was observed between the presence of anti-CarP antibodies and the clinical score. A similar weak correlation was identified between anti-CarP IgG antibodies and anti-collagen IgG antibodies (Fig. 1f).

**Fig. 2:**
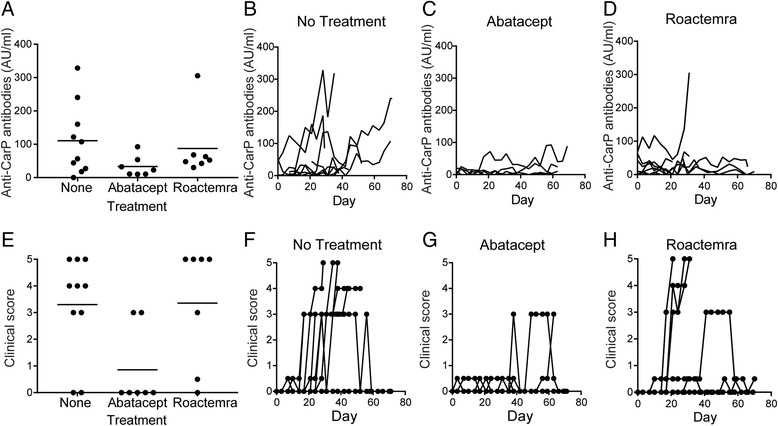
Abatacept treatment reduces the development of anti-citrullinated protein antibodies (anti-CarP) antibodies and affects the severity of collagen-induced arthritis in rhesus monkeys. **a** Anti-CarP IgG antibodies were measured by enzyme-linked immunosorbent assay in rhesus monkeys treated with no medication (n = 10), abatacept (n = 7) or Roactemra (n = 7). The highest anti-CarP antibody level for each monkey is shown. Timelines for the anti-CarP antibodies over time are shown for no treatment (**b**), abatacept (**c**) and Roactemra (**d**). **e** The clinical score is shown in rhesus monkeys treated with no medication (n = 10), abatacept (n = 7) or Roactemra (n = 7). The highest clinical score for each monkey is shown. Timelines for the clinical score over time are shown for no treatment (**f**), abatacept (**g**) and Roactemra (**h**). Day 0 is the time point of immunization with collagen. The data in **e**-**h** were presented before in two separate studies [22, 23] and are shown here as comparison. AU/ml, arbitrary units per millilitre

After the detection and characterization of anti-CarP antibodies in rhesus monkeys, the effect of several (preventive) treatments on these autoantibody levels was investigated as well, using abatacept and Roactemra as model treatments. Lower levels of anti-CarP antibodies were observed in the group treated with abatacept when compared to the control group without treatment (Fig. 2a). Although differences between the abatacept and the no-treatment group were not significant with these small group sizes, a clear trend was visible (Mann-whitney U test, *p* = 0,087). These differences also seemed to be consistent over time, showing an increase in anti-CarP antibodies in the control group, while low levels of anti-CarP antibodies were detected in the abatacept-treated group (Fig. 2b-d). For Roactemra, an increase in anti-CarP antibodies was only seen in one animal, but no statistical differences were observed between Roactemra and the control group when comparing the highest observed level of anti-CarP antibodies (Mann-whitney U test, *p* = 0,89).

Furthermore, the effect of the treatments on the clinical score was investigated. Out of the three treatment groups, abatacept-treated monkeys had a reduced clinical score, when comparing the highest achieved clinical score for each of the animals (Mann-whitney U test, *p* = 0.02) (Fig. 2e). The timelines show that the abatacept-treated animals do not develop full-blown arthritis, while this is not the case for the animals without treatment or for animals treated with Roactemra (Fig. 2f-h). No differences in clinical score were observed between the no-treatment group or the group treated with Roactemra (Mann-Whitney U test, *p* = 0.67).

## Discussion

Here we have shown that anti-CarP antibodies can be present in rhesus monkeys and show that these autoantibodies are especially increased after the induction of CIA. Interestingly, ACPA, which target a very similar post-translational modification as anti-CarP antibodies were not detected in this animal model. In patients with RA, there is a large overlap in the positivity for ACPA and anti-CarP antibodies and detailed studies have been performed to confirm that ACPA and anti-CarP antibody-positive serum contains both cross-reactive and non-cross-reactive antibodies [5]. Now in the context of both mice [16, 19] and monkeys, only anti-CarP reactivity is observed, indicating that in these animals, anti-CarP antibodies are not cross-reactive to citrullinated proteins. In mice, ACPA have also been difficult to detect, while they are prominently present in patients with RA. The notion that ACPA are difficult to detect in both rodents and rhesus monkeys indicates that there is a clear difference between the two autoantibodies. Furthermore, RF was not detected in rhesus monkeys after CIA induction, conforming to previous data using the same model [20]. It is not clear why such discrepancies in autoantibody status are observed between human and animal models. However, these data do indicate that induction of arthritis using collagen injections results in differences in disease development and (sub)clinical presentation when compared to the natural onset of RA in humans.

We observed a clear effect on anti-CarP antibody levels by preventive treatment with abatacept, which is a fusion protein consisting of the CTLA4-domain that can bind to CD80 or CD86 and the IgG1 Fc region [25]. This treatment is currently used in patients with RA when they have failed to respond to one or more disease-modifying anti-rheumatic drug (DMARDs). In previous CIA experiments using abatacept treatment, a general reduction in IgM and IgG antibodies was observed [23], which is in line with the reduction in anti-CarP antibodies.

Roactemra, the other intervention used, is also known under the name tocilizumab and is a monoclonal antibody that targets the IL6-receptor [26]. This is also a receptor that is involved in plasma cell development and might therefore be important for antibody production. As in patients with RA, the monkeys that developed CIA also had an increase in IL-6 levels (data not shown), indicating that this disease mechanism might be similar. It is therefore not clear why the treatment with Roactemra did not have an effect on the clinical score in this model.

Furthermore variation was observed between monkeys in the development of anti-CarP antibodies. However, this could be inherent to the model used in the study, since large variety can also be observed between times of disease onset. It should also be noted that the presence of anti-CarP antibodies did not always correlate with the clinical score. Some monkeys did have a large increase in the levels of anti-CarP antibodies, while no arthritic symptoms were observed. Also, some animals with a higher clinical score did not develop high levels of anti-CarP antibodies. These data indicate that anti-CarP antibody levels are not directly correlated with clinical score.

## Conclusion

Altogether, we conclude that abatacept treatment had a dampening effect on the antibody response in CIA, while also preventing disease development. Furthermore, anti-CarP antibodies, but not ACPA or RF were detected in this animal model. Also, anti-CarP antibodies were present before the actual disease onset as observed in both mice and humans.

## Abbreviations

ACPA: Anti-citrullinated protein antibodies
Anti-CarP: Anti-carbamylated protein
BSA: Bovine serum albumin
Ca-FCS: Carbamylated fetal calf serum
CCP2: Cyclic citrullinated peptide 2
CIA: Collagen-induced arthritis
Cit: Citrullinated
DMARD: Disease-modifying anti-rheumatic drug
ELISA: Enzyme-linked immunosorbent assay
FCS: Fetal calf serum
Fib: Fibrinogen
i.v.: Intravenous
MBP: Myelin basic protein
PBS: Phosphate-buffered saline
RA: Rheumatoid arthritis
RF: Rheumatoid factor
STS: Soft tissue swelling
